# Care plus study: a multi-site implementation of early palliative care in routine practice to improve health outcomes and reduce hospital admissions for people with advanced cancer: a study protocol

**DOI:** 10.1186/s12913-021-06476-3

**Published:** 2021-05-27

**Authors:** Jennifer Philip, Roslyn Le Gautier, Anna Collins, Anna K. Nowak, Brian Le, Gregory B. Crawford, Nicole Rankin, Meinir Krishnasamy, Geoff Mitchell, Sue-Anne McLachlan, Maarten IJzerman, Robyn Hudson, Danny Rischin, Tanara Vieira Sousa, Vijaya Sundararajan

**Affiliations:** 1 Department of Medicine, University of Melbourne, Melbourne, Australia; 2 Palliative Care Service, St Vincent’s Hospital Melbourne, Melbourne, Australia; 3 Palliative Care Service, Royal Melbourne Hospital, Melbourne, Australia; 4 Palliative Care Service, Peter MacCallum Cancer Centre, Melbourne, Australia; 5 Medical School, University of Western Australia and Department of Medical Oncology, Sir Charles Gairdner Hospital, Perth, Australia; 6 Northern Adelaide Local Health Network, Modbury Hospital, Adelaide, Australia; 7 Faculty of Health and Medical Sciences, University of Adelaide, Adelaide, Australia; 8 Faculty of Medicine and Health, The University of Sydney, Sydney, Australia; 9 Department of Nursing and Centre for Cancer Research, University of Melbourne, Melbourne, Australia; 10 Academic Nursing Unit, Peter MacCallum Cancer Centre, Melbourne, Australia; 11 Victorian Comprehensive Cancer Centre, Melbourne, Australia; 12 School of Medicine, University of Queensland, Brisbane, Australia; 13 Medical Oncology, St Vincent’s Hospital Melbourne, Melbourne, Australia; 14 Cancer Health Services Research, University of Melbourne, Melbourne, Australia; 15 Safer Care Victoria, Victoria State Government, Melbourne, Australia; 16 Department of Medical Oncology, Peter MacCallum Cancer Centr, Melbourne, Australia; 17 Centre for Health Policy, Health Economics Unit, University of Melbourne, Melbourne, Australia; 18 Department of Public Health, La Trobe University, Melbourne, Australia

**Keywords:** Palliative care, Integrated care, Implementation, Health services

## Abstract

**Background:**

Current international consensus is that ‘early’ referral to palliative care services improves cancer patient and family carer outcomes. In practice, however, these referrals are not routine. An approach which directly addresses identified barriers to early integration of palliative care is required. This protocol details a trial of a standardized model of early palliative care (Care Plus) introduced at key defined, disease-specific times or transition points in the illness for people with cancer. Introduced as a ‘whole of system’ practice change for identified advanced cancers, the key outcomes of interest are population health service use change.

The aims of the study are to examine the effect of Care Plus implementation on (1) acute hospitalisation days in the last 3 months of life; (2) timeliness of access to palliative care; (3) quality and (4) costs of end of life care; and (5) the acceptability of services for people with advanced cancer.

**Methods:**

Multi-site stepped wedge implementation trial testing usual care (control) versus Care Plus (practice change). The design stipulates ‘control’ periods when usual care is observed, and the process of implementing Care Plus which includes phases of *planning, engagement, practice change and evaluation*. During the practice change phase, *all* patients with targeted advanced cancers reaching the transition point will, by default, receive Care Plus.

Health service utilization and unit costs before and after implementation will be collated from hospital records, and state and national health service administrative datasets. Qualitative data from patients, consumers and clinicians before and after practice change will be gathered through interviews and focus groups.

**Discussion:**

The study outcomes will detail the impact and acceptability of the standardized integration of palliative care as a practice change, including recommendations for ongoing sustainability and broader implementation.

**Trial registration:**

Australian New Zealand Clinical Trials Registry ACTRN 12619001703190. Registered 04 December 2019.

## Background

People living with cancer suffer a significant personal burden of symptoms, and have needs for psychological support, as well as better care and information for their families as they navigate their illness [1, 2]. For example, over 50% of patients with advanced cancer report pain, fatigue and shortness of breath, and their families commonly report anxiety and inadequate assistance as they seek to provide support [3]. Both patients and their families report a preference for more information about the illness, its course and the future more broadly in order to plan and make decisions accordingly [1, 3]. However, specific attention to these needs is variable, with oncologists reporting inadequate time, expertise and clinic space to adequately identify and respond to such needs [4, 5].

People with advanced cancer are high users of health care services. Studies of patients in Victoria, Australia who died with cancer reveal that following a diagnosis of incurable disease, people can expect to have a median of three multiday hospital admissions, and spend a total of 29 days in hospital in their final months of life [6–8]. Most (80%) will die in hospital, including 57% in an acute setting. The high levels of healthcare utilization, including at the end of life, have significant cost implications. A study of costs incurred between the period 2009–2013 determined that the average cost of care per cancer case was $49,733 in the last year of life [9], with 80% of these costs due to hospitalisations [10].

Early palliative care is a high value proposition – where value is defined as outcomes that matter to patients and the cost needed to achieve these outcomes [11]. The goals of palliative care are to improve health outcomes for patients with advanced cancer AND to deliver high quality care at lower cost. A significant evidence-base exists (Table 1) to support the systematic implementation of timely palliative care in routine cancer care to reduce variation in care, improve patient outcomes, and reduce health system burden and cost. These benefits have led a number of professional organisations including the American Society of Clinical Oncologists and European Society of Medical Oncologist to recommend that *inpatients and outpatients with advanced cancer should receive dedicated palliative care services, early in the disease course, concurrent with active treatment.”* [28, 29].

**Table 1:** *Palliative care: A high value proposition where value = patient outcomes achieved relative to cost* [11]

***Patient benefits:*** Randomized control trials involving more than 2500 patients with advanced cancer demonstrate improved QOL, symptoms, mood, communication, satisfaction with care & survival [12–21].	
***Family carer benefits:*** Evidence demonstrates improved health outcomes, mood, and satisfaction and survival [17, 22]	
***Health Service Benefits:*** Earlier palliative care referrals are associated with 70% greater likelihood of death outside hospital - the preferred option of most patients [23], fewer emergency presentations (48% vs 68%; *P* < .001), & hospital admissions (52% vs 86%; P < .001) [24, 25].	
***Reduced cost*:** Evidence from systematic reviews report costs savings of $4251 per patient and simultaneous improvement of quality of care [26]	
***Timing of palliative care is important to realise benefits:*** incremental advantages according to length of engagement with palliative care; > 2 weeks before death associated with reduced hospital death (*P* < 0.001), more than 4 weeks with fewer emergency presentations (≥4 weeks, P < 0.001) [24]. Expert consensus suggests referral should occur ≥3 months before death [27].	

Despite evidence of benefit and recommendations for best practice, only 59% per cent of 29,680 cancer inpatients in Victoria, Australia accessed any palliative care in their last year of life [7]. Of those who did access palliative care, this occurred very late – a median of 27 days before death [7]. For 61%, the first palliative care consultation occurred in the final hospital admission which concluded with death. Patients in this cohort had at least one (83%) or two or more (38%) indicators of poorer quality of end of life care [7], defined as more than 14 days in hospital, more than one acute admission or Emergency Department presentation in the last 30 days of life [30]. Further, while people consistently express a preference to die at home [31], only 20% of Victorian cancer patients in this study died at home. Conversely, in a cohort of people with high grade glioma, those who accessed timely palliative care had a 70% greater chance of death at home [23].

Several barriers to early palliative care integration have been identified and include: (1) patients and clinicians viewing palliative care as care of the imminently dying [32, 33]; (2) uncertainty about when to refer; (3) lack of relationships and confidence in the quality of palliative care service; and (4) capacity of community-based palliative care services to respond to demand [34]. There is growing evidence for the efficacy of outpatient palliative care services in addressing many of these barriers, particularly services that are integrated with oncology outpatient clinics. Notably, outpatient clinics facilitate early engagement with palliative care as they provide access to patients in usual care settings and are ‘culturally’ consistent with ongoing cancer therapy [34]. Outpatient palliative care is also associated with improved satisfaction among clinicians including referring oncologists as they facilitate clinician relationships of trust, expedite engagement with community care, and are proven to be effective with improved patient and health service outcomes [35, 36].

Fundamental to the routine integration of palliative care for patients with advanced cancer is to address clinician uncertainty around timing of palliative care referrals. Transition points – which are readily recognizable, objective points within a cancer illness course, have been endorsed by an international expert Delphi consensus as a key element of standardizing palliative care referrals and thus, reducing variation in care [37]. Previous research on the health care use of Victorian patients with high grade glioma (HGG) and metastatic breast, prostate as well as lung cancers demonstrated clear disease-specific transition points (e.g. multiday admission with metastatic disease) in the illness trajectories, which heralded subsequent increased health service utilisation and poor prognosis [7]. Preliminary data from a current phase 2 randomised control trial (RCT) of early palliative care introduction reveals that definitions of transition points vary across cancer types [38], and therefore a ‘one-size-fits all’ approach to early palliative care in cancer illness cannot be applied. Preliminary data from this study [38] also reveals feasibility and acceptability of early palliative care to both patients and health professionals.

Drawing on the evidence presented thus far, combined with the body of evidence accrued by the research team on the nature of current practice and needs – including evaluations of feasibility and effectiveness of responses [6–8, 32, 33, 38, 39], this study aims to develop and implement a population-based intervention called *Care Plus* to reduce hospital admissions at the end of life – consistent with the preferences of most patients.

The development of this study was guided by the UK Medical Research Council methodological framework for developing complex interventions, and the study objectives will be evaluated in accordance to the core domains of the RE-AIM evaluation framework – Reach, Effectiveness, Adoption, Implementation and Maintenance [40].

### Objectives

Primary Objective: To reduce acute hospitalisation days in the last 3 months of life for people with advanced cancer (Effectiveness).

#### Secondary objectives

1. To improve timely access to early palliative care, defined as first contact at least 90 days before death, for people with advanced cancer (Reach)
2. To improve quality of end of life care for patients with cancer, with reduced acute health system use (Effectiveness)
3. To assess the acceptability of *Care Plus* according to patients, families and healthcare providers (Adoption, Implementation)
4. To assess the fidelity of delivery of *Care Plus* according to core components of the intervention (content, follow-up, GP case conference) (Adoption, Implementation)
5. To assess the impact of *Care Plus* implementation of the total cost of healthcare utilisation (including hospitalisation, Emergency Department presentation, ambulatory care, general practitioner use, medications) in the last 3 months of life (Effectiveness)
6. To develop an Implementation package of recommended approaches and effective strategies to establish *Care Plus* in other settings (Maintenance)

#### Core hypotheses

1. The implementation of *Care Plus* will reduce the acute hospitalisation days in the last 90 days of life by 25% for patients with advanced cancer.
2. The implementation of *Care Plus* will increase by 25% the proportion of patients with advanced cancer referred to palliative care ≥90 days before death.
3. *Care Plus* will decrease by 25% the proportion of patients experiencing more than 1 indicator of poor quality end of life care defined as: (≥ 2 acute hospital admissions, length of stay ≥14 days, ≥2 Emergency Department presentations in the last month of life, chemotherapy in the last 14 days of life, late or absent palliative care referral, and death in acute hospital) [41].
4. *Care Plus* will be acceptable to patients, families and health professionals
5. *Care Plus* will be delivered according to core components detailed (Table 2) in ≥50% of patients.
6. *Care Plus* will reduce the total health system costs incurred in last 3 months of life.
7. An Implementation Package will enable *Care Plus* to be scaled to other health services nationally and with international implications.

**Table 2:** *Core Components of Care Plus*

1. **Palliative care is introduced at cancer-type specific key transition points**	
2. **Initial hospital-based palliative care consultation (as inpatient or outpatient), addressing:** • Review of underlying disease management • Screening for symptom distress • Screening for psychological distress • Review of informal community supports, including local community palliative care • Providing information • Advance care planning discussions • Involvement of family carer, including enquiry of concerns and needs of information	
3. **Regular, prescribed outpatient follow up:** • At minimum monthly for at least 2 months • At conclusion of the prescribed intervention, a clinical decision is made between the patient and palliative care physician for individualized follow-up beyond standard ‘dose’.	
4. **Case conference with the general practitioner within 28 days, addressing:** • Current and anticipated problems • Recommended management and therapies	

## Methods

### Study design

**Fig. 1:**
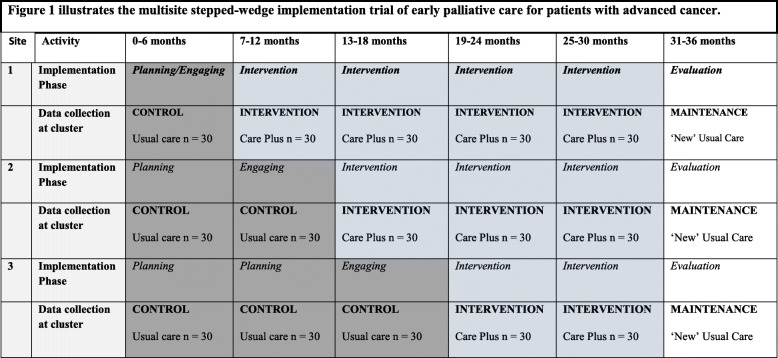
Multisite stepped-wedge implementation trial of early palliative care for patients with advanced cancer

This study is a stepped-wedge implementation trial of early palliative care for patients with advanced cancer (Fig. 1). This design includes a series of phases: (1) control periods when usual care is observed while phases of planning and engagement occur, (2) practice change periods when *Care Plus* is administered, and (3) maintenance periods, when an evaluation phase enables measurement of ongoing impact and sustainability in cancer care.

### Study setting

This study is being undertaken at four metropolitan tertiary cancer services in two Australian states. These hospitals have specialist palliative care providing both inpatient and outpatient consultation services.

### Implementation framework

Consistent with the Consolidated Framework for Implementation Research (CFIR), this study reconciles scientific rigor with the pragmatism required to achieve successful change in complex systems by taking into account multiple dynamics and influences upon the implementation process. Understanding the internal and external context of each clinical site including its structural characteristics, culture, divisions, readiness for implementation and external policies are fundamental to the implementation framework. Equally important are the individuals (including patients and health providers), the characteristics of the intervention and the overall implementation process itself which involves phases of *planning, engagement, practice change and evaluation* [42].

### Implementation phases

It is important to note that while the implementation occurs in phases, this is not a linear process and many activities relevant to each phase may occur concurrently. Consistent with the Consolidated Framework for Implementation Research, the implementation process will be tailored and adapted to the local clinical sites.

### Planning phase

1. Observation and measurement of usual oncological care at the site prior to the implementation of *Care Plus*, including health service use, hospitalisation, cost, palliative care practices (i.e. access, timing, variation across cancer types), and quality of end of life care.
2. Assessment of sector readiness for change, including focus groups/individual interviews with consumers and clinicians exploring:
  - Attitudes to *Care Plus* implementation.
  - Identification of key opinion leaders.
  - Organisational structures and workflow processes that serve as barriers and facilitators.
  - Preferred communication strategies and acceptability of different resource materials.
3. Logistical planning (already tested in pilot study) to ensure ongoing:
  - Palliative care capability and capacity.
  - Clinic supportive structures available.
  - Mechanism to standardize the identification of patients reaching transition points.

### Engagement phase

1. Establishing study support:
  - Identification of key opinion leaders and local advocates including consumer advocates.
  - Seeking endorsement for change from key hospital and policy leaders.
2. Consultation with health providers and consumers at each site to determine:
  - Five advanced cancer types to target for implementation of *Care Plus*. Consistent with the Consolidated Framework for Implementation Research, cancer types will be:
    nominated by key clinicians at each site for prioritisation, which may differ between sites; and
    feasible with minimum 30 patients with advanced disease per cancer type per site annually.
  - Identified and endorsed transition points for each of the identified cancer types
  - Tailored resource materials and delivery processes for each site, while still maintaining the core components of *Care Plus*.
  - Develop patient resources to explain the activities/benefits/role of early palliative care.
3. Training related to *Care Plus* delivery at each site for:
  - Palliative care physicians.
  - Oncology clinicians.
  - Cancer navigators and other staff involved in *Care Plus* processes.

### Implementation of care plus as practice change phase

During the practice change phase, all patients with targeted cancers reaching transition point will, by default, receive *Care Plus* as part of a hospital wide practice change.

A series of cancer-specific standard operating procedures for the implementation of *Care Plus* will be developed, including the steps of:

- Identification of eligible patients reaching ‘transition point’ and process of alerting clinician;
- Discussion with patient and family by treating clinician/clinical team member;
- Provision of written information to patient and family;
- Delivery of *Care Plus* (as operationalised in Table 2):
  Consultation by palliative care physician/clinical team member at or within 6 weeks of transition point.
  Prescribed follow up with details of the consultation recorded using domains of PC-NAT [43].
  General Practitioner Case Conference.

### Evaluation phase

Each of the domains of the RE-AIM Implementation Framework (Reach, Effectiveness, Adoption, Implementation and Maintenance) will be examined using mixed methodologies, consistent with the complex nature of the implementation (Table 3).

**Table 3:** Summary of Evaluation Consistent with RE-AIM and Mapped to Objectives

Objective	Hypothesis	Evaluation domain	Method
1. To reduce acute hospitalisation days for patients with advanced cancer	Implementation of *Care Plus* will reduce the acute hospitalisation days in the last 90 days of life by 25% for patients with advanced cancer.	Effectiveness	Comparison of the outcome variable for patients enrolled during the control versus the practice change periods using linked data from hospital medical records, routinely collected state-based administration datasets (i.e. MBS/PBS^a^), and the death registry.
2. Improve timely access to palliative care	Implementation of *Care Plus* will increase by 25% the proportion of patients with advanced cancer referred to palliative care at least 90 days before death	Reach	
3. Improve quality of end of life care	*Care Plus* will decrease by 25% the proportion of patients experiencing > 1 indicator of poor-quality end of life care	Effectiveness	
4. Assess the acceptability of *Care Plus*	*Care Plus* will be acceptable to patients, families & healthcare providers	Adoption Implementation	Focus groups and individual interviews with patients & healthcare providers
5. To assess the fidelity of *Care Plus* delivery	*Care Plus* will be delivered according to the core elements prescribed in more than 50% of patients	Adoption Implementation	Review of *Care Plus* consultations recorded using PC-NAT^b^ + medical audit of outpatient visits, GP case conferences
6. Assess the impact of *Care Plus* implementation on total cost of healthcare	Implementation of *Care Plus* will reduce total health system costs incurred in last 90 days of life	Implementation Maintenance	Comparison of total health system costs for patients enrolled during the control versus the practice change periods using linked hospital & MBS/PBS data

^a^MBS/PBS Medicare Benefits Schedule provides information of health services provided outside hospitals /Pharmaceutical Benefits Schedule provides information of prescribing outside hospitals in Australia;

^b^PC-NAT – Palliative Care Needs Assessment Tool

### Governance and oversight

The study investigators are supplemented by the establishment of a Community Advisory Committee consisting of consumers, community members and key regional and metropolitan clinicians who give advice and feedback to the investigative team. In addition, clinical sites have key groups who meet regularly and provide input and feedback to the investigators.

Central ethical approval for the trial conduct at all participating sites was provided by the Human Research Ethics Committee at St Vincent’s Hospital Melbourne [HREC 188/19]. The trial was registered with the Australian and New Zealand Clinical Trial Registry [ACTRN12619001703190].

### Funding

Funding for this trial was obtained from the Medical Research Future Fund, NHMRC, Australia via a competitive health services research grant [APP1174028].

### Data collection

This study will collect data from participant cohorts at three key periods during the implementation of *Care Plus* in routine cancer care: (1) pre-practice change, (2) during the practice change period and (3) post-practice change.

1. Pre-Practice Change Period

- Patients with a targeted advanced cancer in receipt of usual care: Prior to implementing *Care Plus*, the study will collect and analyse aggregate data of health service use and cost of health service provision of care for patients with a targeted advanced cancer enrolled in the historical control period, and in receipt of usual oncological care.
- Patients and /or people with current or past experience of a cancer who are able to provide consent will be invited to participate in a focus group/interview to facilitate the development and review of patient and carer resources supporting the implementation of *Care Plus*.
- Clinicians frequently involved in the care of people with a targeted advanced cancer at participating hospital sites and who provide consent will be invited to participate in a focus group/interview with the aim to identify key transition points for the targeted cancers, as well as provide their perspectives on the factors surrounding the sector’s readiness for change.

2. Practice Change Period as *Care Plus* is implemented

- Patients with a targeted advanced cancer attending a participating hospital who reach defined cancer-specific ‘transition point’: In this period, the study will collect and analyse aggregate data of health service use and cost of hospital care for patients with a targeted advanced cancer enrolled in the practice change period, and in receipt of *Care Plus*.

- Key hospital staff including clinicians, administrators and managers who are involved in the implementation of Care Plus at participating hospital sites and who provide consent will be invited to participate in a focus group/individual interview with the aim to identify any barriers and facilitators experienced during the practice change period, as well as their experiences of the practice change to date.

3. Post-Practice Change Period

- Patients in receipt of *Care Plus* and who provide consent will be invited to participate in an interview to provide their perspectives and experiences of *Care Plus* as part of routine cancer care.
- Clinicians involved in the delivery of *Care Plus* and who consent will be invited to participate in a focus group/interview to provide their perspectives and experiences of the practice change.

### Study procedures for data collection

Data Linkage cohort study: Data for the quantitative analysis will come from linked emergency department, hospital discharge, ambulatory visit, pharmaceutical claim and death registration records. These records are held by individual hospitals, state health information units and the Australian Institute of Health and Welfare. Standard processes will be followed to request linked data.

A review of PC-NATs recorded during *Care Plus* consultations, as well as a medical record audit of outpatient visits and GP case conferences will be conducted and analysed to evaluate the fidelity of *Care Plus* delivery.

Qualitative data collection: Qualitative data collection occurring at the three key nominated periods of pre-, during practice change and post-practice change will follow similar procedures. In the pre-practice period, people with experience of cancer will be contacted by email invitation distributed through consumer representatives and managers of the hospitals, and state-based consumer organisations. Those interested in participating will contact the representative or study coordinator and a mutually convenient time will be established for the focus group/interview.

A total of 10–15 individual interviews with patients in receipt of *Care Plus* will be conducted at each of the participating sites. These patients will be identified by a clinical site team member who will initially provide a brief background to the study. Those who express interest in further information will be introduced to the study team, and if indicated, on provision of more detail be invited to participate in an interview at a place and time of convenience.

Clinicians in the pre- during-, and post-practice change period will be recruited via an email invitation distributed via their service communication networks. Those interested will be invited to make contact with the study coordinator and a time for the focus group/individual interview will be organized at the clinical site.

Following study explanation, all willing participants will provide written informed consent. The focus group/interview will follow a semi-structured format (Appendix 1). The discussions will be audio-recorded and transcribed verbatim, and de-identified.

### Planned data analysis

#### Quantitative data analysis

##### Power calculation

Analysis of Victorian data demonstrates that median length of acute hospital stay for advanced cancer patients in the last 90 days of life is 28 days, and the primary objective is to reduce the acute hospitalisation days in the last 90 days of life by 25% for patients with advanced cancer. This corresponds to approximately 6 days and is the average length of stay of a hospital admission. Length of stay in Victorian data is not normally distributed; transformation to the square root normalises it on inspection of a histogram. Analysis will be conducted at a system level (data described below). Assuming an intracluster correlation (ICC) of 0.01, a two-sided alpha of 0.05, a cell size of 30 participants provides > 99% power to detect a difference of 6 days. Varying the ICC from 0.001–0.1 whilst holding all other assumptions constant does not appreciably change the power. This translates to a total sample in the control and intervention periods of 450.

##### Analysis

Description of individual and cell cluster characteristics to assess balance between control and practice change periods will be conducted using means (standard deviations) or median (intraquartile range) for continuous factors and frequencies (percentiles) for categorical variables. To assess the significance of differences in receipt of timely palliative care, a logistic regression model will be fitted with a random effect for cluster and a fixed effect for each step. Further analyses will assess for potential differences in 1) effect size of the practice change periods by site; and 2) temporal decrease in the proportion of participants accessing care during the successive practice change/maintenance periods. For other quantitative outcome variables, a similar analytic approach will be used. Analyses will be undertaken in Stata V15 (Stata Corp, College Station, Tx, USA) and level of significance set at 5%. Analyses will be conducted on an intention-to-treat basis, with reporting consistent with CONSORT guidelines.

##### Economic

Health resource costs will be observed longitudinally (up to death) for patients enrolled during the *Care Plus* practice change periods compared with patients enrolled during control periods. Costs of *Care Plus* implementation will be estimated to account for additional resources including: engagement and training of staff; and development, maintenance/ monitoring. Health utilization and the costs of hospital admissions, emergency department presentations, general practitioner and specialist outpatient clinics (including palliative care), the use of prescription medicines, and the use of community-based palliative care and nursing services will be included in the costing summary. Standard parametric techniques will estimate the 95% confidence limits and *p*-values for the differences in the mean values of the costs.

#### Qualitative data analysis

These data will be analysed according to the principles of iterative thematic analysis, whereby analysis occurs throughout the research process and conducted simultaneously with data collection and final write-up. First stage analysis will involve careful reading of the de-identified transcripts by two members of the research team, identifying broad ideas, issues and concepts emerging from the text. The second stage analysis will involve identifying themes, sub-themes, and relationships between these, with further refinement through discussions with the research team.

## Discussion

As cancer incidence rises, cost-effective models-of-care that improve quality and equity of care for patients and families are increasingly critical. Despite treatment breakthroughs [29], advanced cancer-related deaths occur daily in Victoria, Australia and 8.2 million deaths annually worldwide. [44] Those with advanced disease report high needs and distress that are inadequately addressed. Meanwhile, palliative care which explicitly addresses these concerns [14, 15, 21, 23, 45–47], is accessed variably and late [23, 48, 49]. *Care Plus* is a system wide response which addresses the barriers to engagement with palliative care and implements a practice change to all patients with targeted cancers, thereby reducing variability and improving the standardization of delivery of quality care.

The impact of this population-based, system wide approach will correspondingly be measured by population-based outcomes, namely hospitalization patterns. Yet the finer detail of the impact of *Care Plus* as a service change will be captured using the granular qualitative data available at each clinical site. Consistent with the implementation methodology, we anticipate differences between sites including different challenges and successes.

Ultimately, we seek to provide longevity of the system wide changes by embedding consumer and clinician ownership of the health practices of *Care Plus*. The opportunities to translate these practices to other settings and services will be made available through the results of this study.

## Data Availability

Not applicable.

## Appendix

Interview Guide for Pre-Practice Change Period

**Table:**

Clinician Guiding Questions	Consumer Guiding Questions
1. What are the challenges associated with introducing palliative care? 2. Based on evidence that early palliative care improves patients’ symptoms and quality of life, improve family outcomes and reduce hospitalisation and cost at end of life, our study aims to integrate early palliative care in routine cancer practice for the following five cancers [name nominated cancers for that site]. Given this, when would be the best time to start palliative care for these cancers and how would you perceive we identify patients? 3. What would be helpful for you to ensure access to this care pathway is seamless for patients? 4. Would you be willing to introduce this care pathway and how would you see this happening?	1. What have you heard about palliative care? 2. Do you think there are concerns held by the community about palliative care? What might these be? 3. [provide evidence – and provide background of project] 4. What would you want to know from clinicians about palliative care in routine cancer practice? 5. How would you like information about palliative care to be delivered by your clinician? 6. What other information/guidance would be helpful to cancer patients and carers?

Interview Guide for Practice Change Period

**Table:**

Key hospital staff guiding questions
1. How do you think the new practice change is going? a. What is going well? b. What can be changed so it can work (more) effectively in your team/department?
2. What, if anything, is the practice change altering how you coordinate patient care with other departments in the hospital? a. Has this changed since the implementation commenced?
3. a. [If yes] Can you describe this? b. [If no] Why not?
4. What kind of support or actions (from leaders in your department/or the implementation team) would help make the implementation of Care Plus successful and sustainable?

Interview Guide in the Post-Practice Change Period

**Table:**

Clinician Guiding Questions	Patient Guiding Questions
1. What was your experience of delivering this new care pathway? 2. What went well? 3. Did you encounter any difficult/tricky situations? 4. Did we get the transition point right? 5. What would you recommend moving forward?	1. What was your experience of the *Care Plus* service? 2. What elements of the care service worked well for you? 3. What elements of the care service could be improved? 4. Is there anything else you would like to discuss that we haven’t touched on?
